# Clinically relevant updates of the HbVar database of human hemoglobin variants and thalassemia mutations

**DOI:** 10.1093/nar/gkaa959

**Published:** 2020-10-30

**Authors:** Belinda M Giardine, Philippe Joly, Serge Pissard, Henri Wajcman, David H K. Chui, Ross C Hardison, George P Patrinos

**Affiliations:** The Pennsylvania State University, Center for Computational Biology and Bioinformatics, University Park, PA, USA; Biochimie des pathologies érythrocytaires, Laboratoire de Biochimie et Biologie Moléculaire Grand-Est, Groupement hospitalier Est, Hospices Civils de Lyon, Bron, France; Laboratoire Interuniversitaire de Biologie de la Motricité (LIBM) EA7424, Equipe “Biologie vasculaire et du globule rouge’’, Université Claude Bernard Lyon 1, COMUE Lyon, France; Assistance Publique Hopitaux de Paris), Department of Genetics GHU (Groupe Hospitalier Universitaire Henri Mondor) H. Mondor and Institut Mondor de Recherche biomedicale - INSERM U955 eq2, Creteil France; INSERM U955, CHU Henri Mondor, Creteil, France; Boston University School of Medicine, Department of Medicine, Pathology and Laboratory Medicine, Boston, MA, USA; The Pennsylvania State University, Center for Computational Biology and Bioinformatics, University Park, PA, USA; Department of Biochemistry and Molecular Biology, The Pennsylvania State University, University Park, PA, USA; University of Patras, School of Health Sciences, Department of Pharmacy, Laboratory of Pharmacogenomics and Individualized Therapy, Patras, Greece; Erasmus University Medical Center Rotterdam, Faculty of Medicine and Health Sciences, Department of Pathology, Bioinformatics Unit, Rotterdam, the Netherlands; United Arab Emirates University, College of Medicine and Health Sciences, Department of Pathology, Al-Ain, UAE; United Arab Emirates University, Zayed Center of Health Sciences, Al-Ain, UAE

## Abstract

HbVar (http://globin.bx.psu.edu/hbvar) is a widely-used locus-specific database (LSDB) launched 20 years ago by a multi-center academic effort to provide timely information on the numerous genomic variants leading to hemoglobin variants and all types of thalassemia and hemoglobinopathies. Here, we report several advances for the database. We made clinically relevant updates of HbVar, implemented as additional querying options in the HbVar query page, allowing the user to explore the clinical phenotype of compound heterozygous patients. We also made significant improvements to the HbVar front page, making comparative data querying, analysis and output more user-friendly. We continued to expand and enrich the regular data content, involving 1820 variants, 230 of which are new entries. We also increased the querying potential and expanded the usefulness of HbVar database in the clinical setting. These several additions, expansions and updates should improve the utility of HbVar both for the globin research community and in a clinical setting.

## INTRODUCTION

Hemoglobinopathies are the most common single-gene genetic disorders in humans, resulting from pathogenic genomic variants in the human α-like and β-like globin gene clusters (1). The human α-globin gene cluster is comprised of the *HBZ* (OMIM# 142310), *HBA2* (OMIM# 141850), *HBA1* (OMIM# 141800), *HBM* (OMIM# 609639) and *HBQ1* (OMIM# 142240) genes, which encode the ζ-, α2-, α1- and possibly μ- and θ-globin polypeptide chains, respectively. The human β-globin gene cluster is comprised of the *HBE1* (OMIM# 142100), *HBG2* (OMIM# 142250), *HBG1* (OMIM# 142200), *HBD* (OMIM# 142000) and *HBB* (OMIM# 141900) genes, which encode the ϵ-, ^G^γ, ^A^γ-, δ- and β-globin polypeptide chains, respectively. Many hemoglobin variants result from single nucleotide variants or indels, leading to amino acid replacements, while deleterious variants in either regulatory or coding regions of the human *HBA2*, *HBA1*, *HBB* or *HBD* genes can minimally or drastically reduce their expression, leading to α-, β- or δ-thalassemia respectively.

The HbVar database of hemoglobin variants and thalassemia mutations is one of the oldest and most widely used locus-specific databases (LSDBs), not only from the globin but also from the wider genetic database community. HbVar was launched 20 years ago, in 2001. It was built from previous compilations of variants in books (2,3), converting this information into a publicly available LSDB to provide timely information to interested users, e.g. the globin research community, patients and their parents, and providers of genetic services and counseling. HbVar was developed in such a way to allow for regular data entry updates and corrections, as new hemoglobin variants and thalassemias continue to be discovered. In addition, with a comprehensive query interface, HbVar enables the user to easily access the stored information particularly for the research community, but it is also an aid for physicians in diagnosis. Since its launch, HbVar has rapidly become an important data resource for the globin research community and is considered to be one of the premier LSDBs available to date (4).

Here, apart from the regular data content updates and corrections, we report important new updates in HbVar structure and functionality, aiming both at increasing the impact of the database among not only the globin research but also the clinical community, and facilitating data querying and output.

## UPDATES TO EXISTING DATA

Since the launch of HbVar (5) and the previous database updates in 2004 (6), 2007 (7) and 2014 (8), HbVar information has been expanded by more than 230 additional entries and data corrections, made continually by the database curators. Importantly, Dr. Philippe Joly (Hôpital Edouard Herriot, Unité de Pathologie Moléculaire du Globule Rouge, Lyon, France) and Dr Serge Pissard (Mondor Institute of Biomedical Research, Department of Genetics, Creteil, France) have recently joined the HbVar team as data curators. In order to identify new hemoglobin variants and thalassemia mutations not previously documented in the database, we manually scanned articles from the specialized journal *Hemoglobin*, which frequently publishes new hemoglobin variants and thalassemia mutations, and where applicable, previously undocumented variants and additional information for existing variants have been entered into HbVar. We also benefit from continuous communication with the globin research community and independent researchers, who provide information and references that our curators use both to update the HbVar database content with novel variants and also to rectify data errors and inconsistencies in existing variants.

## THE NEW HbVar HOME PAGE

In order to better capture the data content, interrelated databases and recent updates and user statistics, the HbVar home page has been completely rebuilt. Firstly, the HbVar logo has been redesigned to capture the original concept as well as the Hb molecule notion in a more vibrant manner. Secondly, the query the database functionality now occupies a more central arrangement in the database to facilitate activity by the end-user, compared to the previous situation. Also, we included, in a tabular format, links to important HbVar functionalities and features that are grouped in different rows in the table, such as:

- the **main HbVar functionalities**, e.g. the summary of mutation categories, query of compound heterozygotes phenotype (see next paragraph), most recent updates and frequently asked questions,
- **interrelation with other databases and resources**, such as FINDbase [http://www.findbase.org; (9)], the Leiden Open-Access Variation database [LOVD; http://www.lovd.nl; (10)], dbSNP [https://www.ncbi.nlm.nih.gov/snp; (11)] and the Penn State Genome Browser, which is a mirror of the UCSC Genome Browser [https://genome.ucsc.edu; (12)] customized to present data from HbVar and other resources.
- **auxiliary information**, such as the SNP coordinate converter (see below), reference sequences, and a widely used chart with mass differences resulting from amino acid substitutions.

HbVar curators and contact information are provided at the end of the new HbVar home page.

## CLINICALLY RELEVANT QUERY PAGE UPGRADES

HbVar database has been considered a beneficial resource in hemoglobin research since its establishment. As such, since its last update, we opted to focus on clinically relevant updates that would also make HbVar more useful to the clinical community as well. Below, we describe two new features that aim to help clinicians in better exploiting the wealth of information available in HbVar. Both features are self-explanatory with a brief description at the top of each query window to facilitate the user.

### Compound heterozygotes phenotype

Given the many genomic variants that yield different Hb variants and thalassemia mutations, and most of them in high allelic frequencies (6,9), there are often compound heterozygous cases that have different clinical features and laboratory findings (13). Knowing the specific clinical features of a combination of certain variants is crucial to establish accurate diagnosis. For example, a common misdiagnosis can be the combination of an *HBB* and an *HBD* gene variant that leads to normal HbA_2_ levels. The normal levels of HbA_2_ means that these cases can easily escape the attention of the physician but identifying them can be of utmost importance especially in the case of prenatal diagnosis.

**Figure 1.**
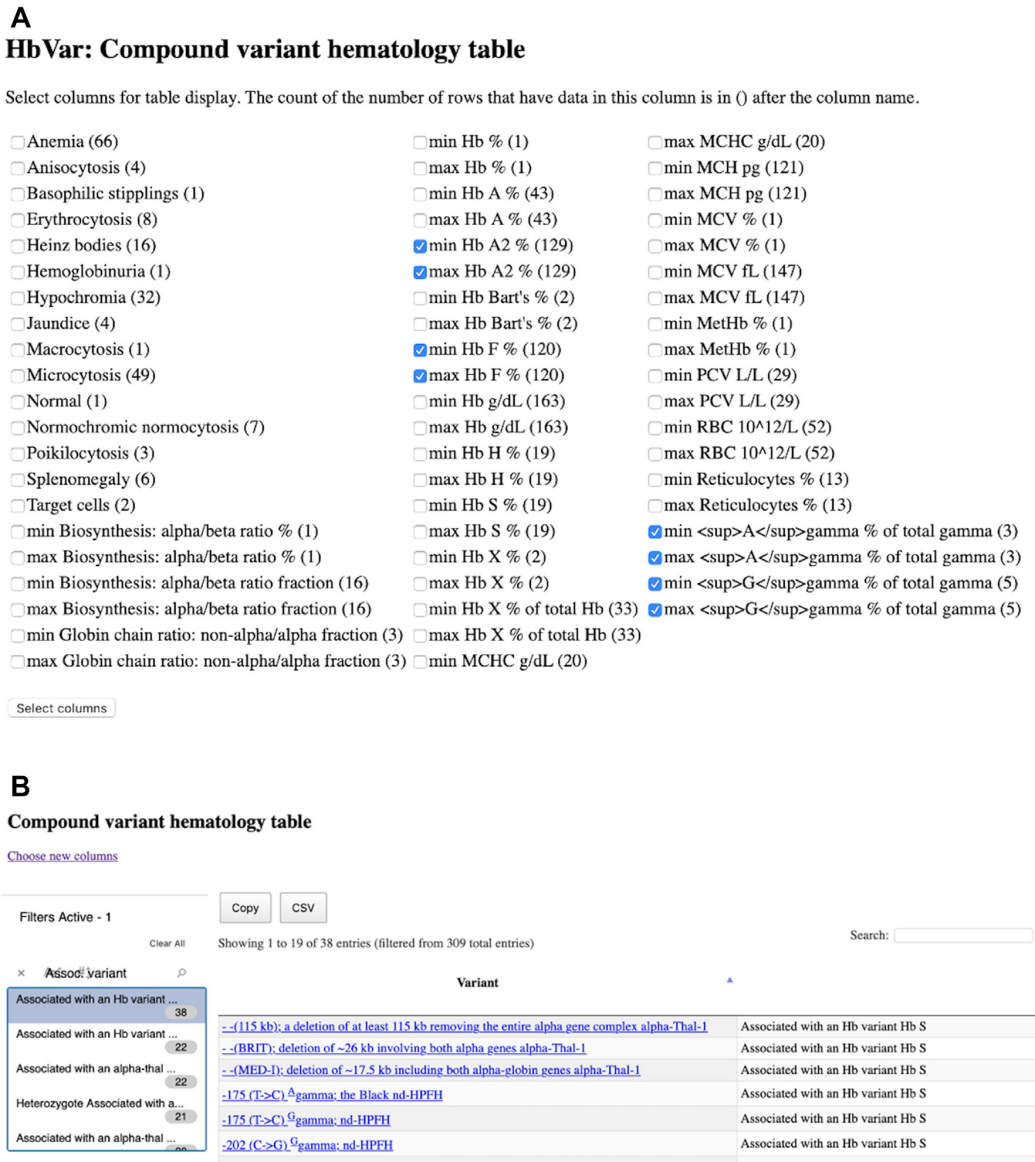
**The new compound heterozygotes phenotype tool of HbVar**. A user can select the desired columns for the table output display from a wide variety of options, according to the available HbVar data. The count of the number of rows that have data in each column is provided in brackets after each column name (**A**). Upon selection of the columns, a table is generated and the user can narrow down his search by selecting the data from the filters at the left side of the page (**B**).

Therefore, we developed a tool to allow the HbVar users from the clinical community to explore the clinical features associated with combinations of globin gene alleles in compound heterozygotes (a total of 309 entries of the database). The compound heterozygotes phenotype tool (available at http://globin.bx.psu.edu/cgi-bin/hbvar/hematable) includes a large menu of clinical features from which the user can select by ticking on the respective boxes (Figure 1). The selected features will be included as columns in the subsequent table generated by clicking on the ‘Select columns’ button. The first two columns of the table include the globin gene alleles combination for all 309 HbVar entries with information for compound heterozygotes. The menu at the left side of the screen includes filters that allows the user to narrow down their query, the top one of which is the associated variant with the number of entries for this variant in brackets. For example, the user can select the entries in which Hb S is the associated allele, where the query returns 38 results and explore the clinical features that he has previously selected in the table output. Each HbVar entry is a hyperlink that takes the user to the respective HbVar entry page (Figure 1). The query output can be also exported in a .csv file format. Lastly, the user can alter the composition of the table by selecting new columns by clicking on the button at the top left corner of the page.

### SNP coordinates converter

With the different numbering systems to determine a genomic position, there is often ambiguity as to the position of a specific variant, especially among clinicians who often need urgently to assess clinical information of a specific variant. We have therefore developed a tool that provides this positional information and specifically converts the genomic position provided in the common number system to the various other systems, such as the official Human Genome Organization (HUGO) genomic DNA-based description, the Human Genome Variation Society (HGVS) coding DNA reference sequence, the DNA-based description using the GenBank reference sequences NG_000007.3 and NG_000006.1 and lastly, the common protein-based description. This tool is available at http://globin.bx.psu.edu/cgi-bin/hbvar/coorSeqCheck.

**Figure 2.**
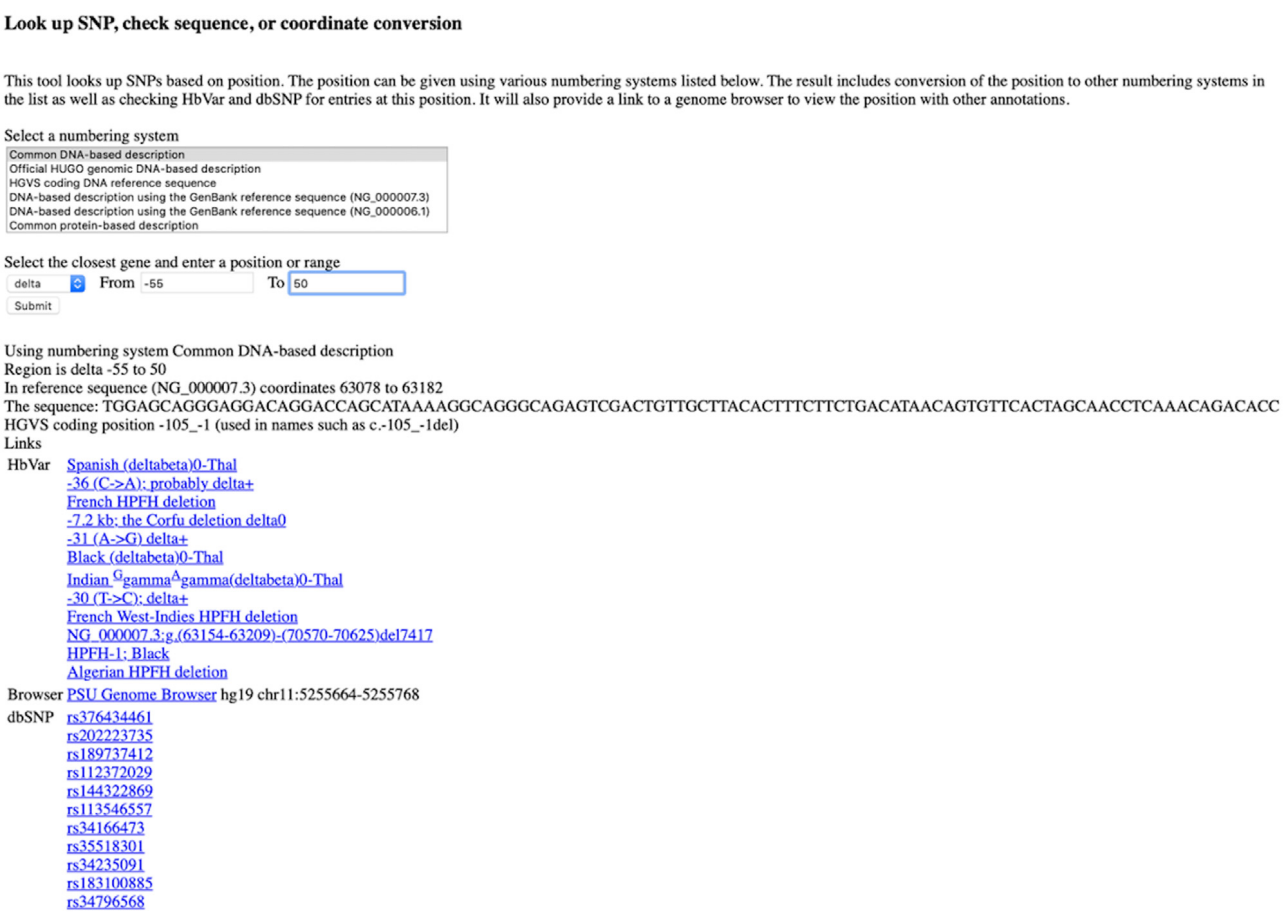
**The** **new** **SNP** **coordinate** **converter** **tool**. This tool looks up SNPs based on their position in the DNA or protein that can be given using various numbering systems (see text). The result includes conversion of the position or range to other numbering systems in the list as well as checking HbVar and dbSNP for entries at this position or range, respectively. It also provides a link to a genome browser to view the position with other annotations.

In the demo query available in Figure 2, the user can select a given position or range for a specific globin chain (in this case the range between −50 and +50 for the delta globin chain, using the common DNA-based description. By clicking on the ‘Submit’ button, the query returns 12 HbVar entries and 11 dbSNP entries, from the PSU Genome browser, along with the synonyms of these genomic positions in all other numbering systems, provided at the top of the page.

## DATABASE ACCESS

Since their launch in January 2001, the HbVar database and associated resources at the Globin Gene Server [http://globin.bx.psu.edu], such as the online Syllabi, are regularly used worldwide. Also, HbVar is very frequently accessed by Facebook and mobile devices. Users frequently contact the curators and the rest of the HbVar team members in order to submit new hemoglobin variants and/or thalassemia mutations, report missing information for existing mutants, identify inconsistencies and/or erroneous entries, and even propose collaborative projects related to HbVar data records.

Since its last update, and as seen in the ‘User statistics’ page that is now available (http://globin.bx.psu.edu/hbvar/usage_graphs.html), the number of annual users now exceeds 15000 for the query page and 8000 for the Summary page (based on unique IP addresses). These figures show the utility of HbVar for the globin research community.

## FUTURE PROSPECTS

HbVar has become, since its inception and first launch, a key data resource for information about DNA variants leading to hemoglobinopathies and is still considered one of the most important LSDBs from the various existing ones. Key factors that have contributed to its broad adoption and success are (a) its constant data update and improvements, mostly driven by the long-term devotion and enthusiasm of the data curators and other researchers involved in this project, coming both from Europe and the US, (b) its dynamic data querying and visualization tools, in conjunction with the UCSC and PSU genome browsers, that are constantly being upgraded to become more user friendly and (c) its interrelation with other stable and well-respected international databases. All these features allowed HbVar to maintain a positive impact on the research community and also allowed to attract funding on a continuous basis, dedicated or related to other projects. This is particularly important for keeping HbVar operational, in an environment where dedicated funding opportunities for database development and curation are often very hard to secure, frequently resulting in the discontinuation of many useful databases.

In order to ensure continuous HbVar data enrichment, we plan to implement a broader data searching strategy that includes text-mining tools and other electronic search procedures. This will complement the already existing tight links to the scientific journal *Hemoglobin* and also other resources such as the Human Gene Mutation database (www.hgmd.org.uk; (14)), next to existing databases with which HbVar has already existing bidirectional links (7,8).

The recent emphasis that HbVar has given to expand its impact also among clinicians apart from researchers involved in globin research highlights its potential to make an impact in the clinical globin community, as well. In particular, HbVar can constitute a focal point for genotype and phenotype data collection from a very large number of hemoglobinopathy patients in registries and clinics worldwide. Similar to the CFTR2 project (www.cftr2.org; (15), such long-term effort would entail a thorough genotype and clinical phenotype data contribution, based on the already well-documented microattribution approach (16,17), allowing the identification of rare variants associated with disease. In these individuals, 159 *CFTR* gene variants had an allele frequency of 0.01%. These variants were evaluated for both clinical severity and functional consequence, with 127 (80%) meeting both clinical and functional criteria consistent with disease. Assessment of disease penetrance in 2,188 fathers of individuals with cystic fibrosis enabled assignment of 12 of the remaining 32 variants as neutral, whereas the other 20 variants remained of indeterminate effect. This study illustrates that sourcing data directly from well-phenotyped subjects can address the gap in our ability to interpret clinically relevant genomic variation.

## References

[1] Weatherall D.J., Clegg J.B. The Thalassaemia Syndromes. 4th edn Wiley-Blackwell.

[2] Huisman T.H.J., Carver M.F., Baysal E. A Syllabus of Thalassemia Mutations. 1997; Augusta The Sickle Cell Anemia Foundation.

[3] Huisman T.H.J., Carver M.F., Efremov G.D. A Syllabus of Human Hemoglobin Variants, 2nd edn. 1998; Augusta The Sickle Cell Anemia Foundation.

[4] Mitropoulou C., Webb A.J., Mitropoulos K., Brookes A.J., Patrinos G.P. Locus-specific databases domain and data content analysis: Evolution and content maturation towards clinical use. Hum. Mutat. 2010; 31:1109–1116. 20672379 10.1002/humu.21332

[5] Hardison R.C., Chui D.H., Giardine B., Riemer C., Patrinos G.P., Anagnou N., Miller W., Wajcman H. HbVar: a relational database of human hemoglobin variants and thalassemia mutations at the globin gene server. Hum. Mutat. 2002; 19:225–233. 11857738 10.1002/humu.10044

[6] Patrinos G.P., Giardine B., Riemer C., Miller W., Chui D.H., Anagnou N.P., Wajcman H., Hardison R.C. Improvements in the HbVar human hemoglobin variants and thalassemia mutations for population and sequence variation studies. Nucleic Acids Res. 2004; 32:D537–D541. 14681476 10.1093/nar/gkh006 PMC308741

[7] Giardine B., van Baal S., Kaimakis P., Riemer C., Miller W., Samara M., Kollia P., Anagnou N.P., Chui D.H., Wajcman H. et al. HbVar database of human hemoglobin variants and thalassemia mutations: 2007 update. Hum. Mutat. 2007; 28:206. 10.1002/humu.9479 17221864

[8] Giardine B., Borg J., Viennas E., Pavlidis C., Moradkhani K., Joly P., Bartsakoulia M., Riemer C., Miller W., Tzimas G. et al. Updates of the HbVar database of human hemoglobin variants and thalassemia mutations. Nucleic Acids Res. 2014; 42:D1063–D1069. 24137000 10.1093/nar/gkt911 PMC3964999

[9] Kounelis F., Kanterakis A., Kanavos A., Pandi M.T., Kordou Z., Manusama O., Vonitsanos G., Katsila T., Tsermpini E.E., Lauschke V.M. et al. Documentation of clinically relevant genomic biomarker allele frequencies in the next-generation FINDbase worldwide database. Hum. Mutat. 2020; 41:1112–1122. 32248568 10.1002/humu.24018

[10] Fokkema I.F., Taschner P.E., Schaafsma G.C., Celli J., Laros J.F., den Dunnen J.T. LOVD v.2.0: the next generation in gene variant databases. Hum. Mutat. 2011; 32:557–563. 21520333 10.1002/humu.21438

[11] Sayers E.W., Beck J., Brister J.R., Bolton E.E., Canese K., Comeau D.C., Funk K., Ketter A., Kim S., Kimchi A. et al. Database resources of the National Center for Biotechnology Information. Nucleic Acids Res. 2020; 48:D9–D16. 31602479 10.1093/nar/gkz899 PMC6943063

[12] Lee C.M., Barber G.P., Casper J., Clawson H., Diekhans M., Gonzalez J.N., Hinrichs A.S., Lee B.T., Nassar L.R. et al. UCSC Genome Browser enters 20th year. Nucleic Acids Res. 2020; 48:D756–D761. 31691824 10.1093/nar/gkz1012 PMC7145642

[13] Patrinos G.P., Antonarakis S.E. Speicher M., Antonarakis S.E., Motulsky A. Human Hemoglobin. Human Genetics: Problems and Approaches. 2010; 4th edn Heidelberg Springer 365–401.

[14] Stenson P.D., Mort M., Ball E.V., Chapman M., Evans K., Azevedo L., Hayden M., Heywood S., Millar D.S., Phillips A.D. et al. The Human Gene Mutation Database (HGMD(®)): optimizing its use in a clinical diagnostic or research setting. Hum. Genet. 2020; 139:1197–1207. 32596782 10.1007/s00439-020-02199-3 PMC7497289

[15] Sosnay P.R., Siklosi K.R., Van Goor F., Kaniecki K., Yu H., Sharma N., Ramalho A.S., Amaral M.D., Dorfman R., Zielenski J. et al. Defining the disease liability of variants in the cystic fibrosis transmembrane conductance regulator gene. Nat. Genet. 2013; 45:1160–1167. 23974870 10.1038/ng.2745 PMC3874936

[16] Patrinos G.P., Cooper D.N., van Mulligen E., Gkantouna V., Tzimas G., Tatum Z., Schultes E., Roos M., Mons B. Microattribution and nanopublication as means to incentivize the placement of human genome variation data into the public domain. Hum. Mutat. 2012; 33:1503–1512. 22736453 10.1002/humu.22144

[17] Giardine B., Borg J., Higgs D.R., Peterson K.R., Philipsen. S, Maglott D., Singleton B.K., Anstee D.J., Basak A.N., Clark B. et al. Systematic documentation and analysis of human genetic variation in hemoglobinopathies using the microattribution approach. Nat. Genet. 2011; 43:295–301. 21423179 10.1038/ng.785 PMC3878152

